# Transantral Extraction of an Ectopic Third Molar Due to Suspected Pathology

**DOI:** 10.7759/cureus.117225

**Published:** 2026-09-30

**Authors:** George Hauser, Carson N Van Etta, Trevor Miller, Jonathan Voge, John Kim

**Affiliations:** 1 Oral and Maxillofacial Surgery, William Beaumont Army Medical Center, El Paso, USA; 2 Oral and Maxillofacial Pathology, William Beaumont Army Medical Center, El Paso, USA

**Keywords:** antrum, endoscope, maxilla, odontoma, rigid fixation

## Abstract

Ectopic eruption of teeth in the maxillary sinus is an uncommon phenomenon, with third molars the most frequently involved. Teeth affixed to the posterior sinus wall represent a particularly rare anatomical variant with unique surgical challenges. A 21-year-old male presented with an ectopic maxillary third molar (#1) affixed to the posterior wall of the right maxillary sinus. The tooth was successfully removed with endoscope assistance, and the anterior wall of the maxillary sinus was replaced and secured with a 0.8 mm fixation plate. The patient recovered without incident. This report adds another treatment option to the management of a rare occurrence of an ectopically erupted wisdom tooth within the maxillary sinus. It can also be utilized for any foreign body in the antrum (FBITA).

## Introduction

Ectopic eruption of teeth in the maxillary sinus is an uncommon developmental anomaly. Most commonly, the culprits are maxillary third molars [1,2]. The aetiology of this phenomenon remains multifactorial, including developmental disturbances, such as cleft palate, displacement of the tooth by trauma or pathology, infection, genetic factors, or dental crowding; however, most cases are idiopathic [2]. Most ectopic teeth in the maxillary sinus are asymptomatic and discovered incidentally on routine radiographic surveillance. Symptomatic cases may present with chronic sinusitis, purulent discharge, facial pain, headaches, or ophthalmic complications [2-4].

The location of ectopic teeth within the maxillary sinus varies considerably [1]. Teeth affixed to the posterior sinus wall represent a particularly challenging anatomical variant due to their proximity to critical structures and the technical difficulty of surgical access [1]. While panoramic radiography serves as the initial diagnostic tool, cone beam computed tomography has become the gold standard for precise localization of ectopic teeth and assessment of associated pathology prior to surgical intervention [1,5].

Management of ectopic teeth in the maxillary sinus requires surgical removal to prevent complications, including chronic sinusitis, cyst formation, and potential malignant transformation [1,6]. Traditional surgical approaches include the Caldwell-Luc technique, while other literature describes minimally invasive endoscopic approaches and trans-posterolateral wall techniques for teeth in challenging locations [2,7-9]. The choice of surgical approach depends on tooth location, size, presence of associated pathology, and surgeon expertise [7].

We present the case of a 21-year-old male of South Asian descent with an ectopic maxillary third molar affixed to the posterior wall of the right maxillary sinus. This case highlights the diagnostic workup, surgical considerations, reconstruction of the access, and management of this rare anatomical variant.

## Case presentation

We report the case of a 21-year-old male of South Asian ancestry with a history of treated chronic demyelinating polyneuropathy who presented with an ectopic maxillary third molar (#1) affixed to the posterior wall of the right maxillary sinus. The foreign body in the antrum (FBITA) was initially discovered on routine orthopantomographical screening (Figure 1), and the patient was referred to the oral and maxillofacial surgery clinic for evaluation and treatment. The patient denied any of the usual sinus symptoms associated with FBITA, including sinusitis, congestion, feeling of fullness, other odontogenic discomforts, orbital changes, and reported full V2 sensation. Additional CT imaging resulted in an impression of an ectopic tooth in the right maxillary sinus (Figure 2). At this point, the differential diagnosis included odontoma; the recommendation was made to the patient for removal of the FBITA along with microscopic histopathological analysis. The patient agreed and desired removal of all four wisdom teeth, including the FBITA, #1.

**Figure 1:**
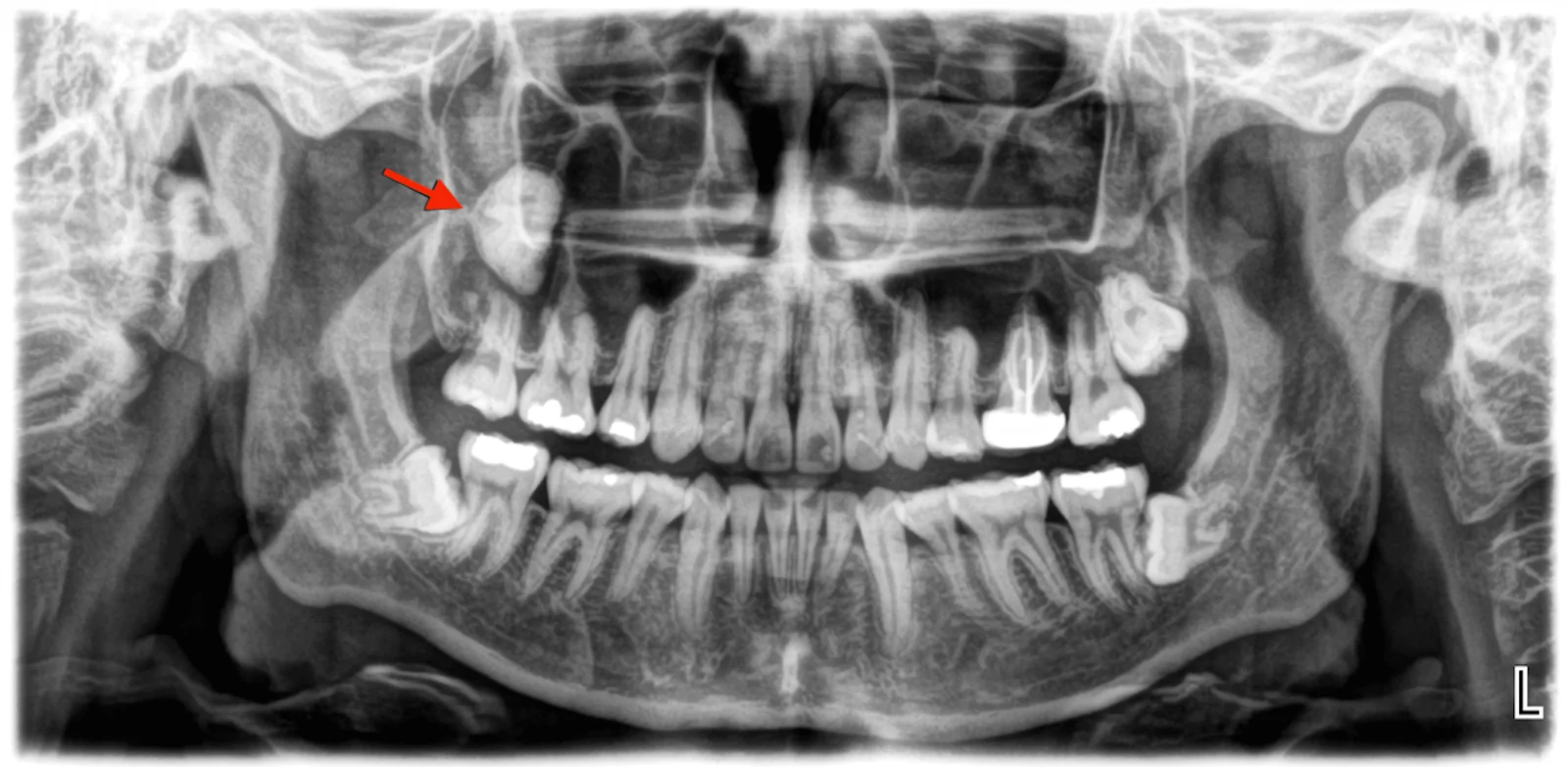
Preoperative orthopantomogram demonstrating a radiopacity superior to the right second molar (arrow).

**Figure 2:**
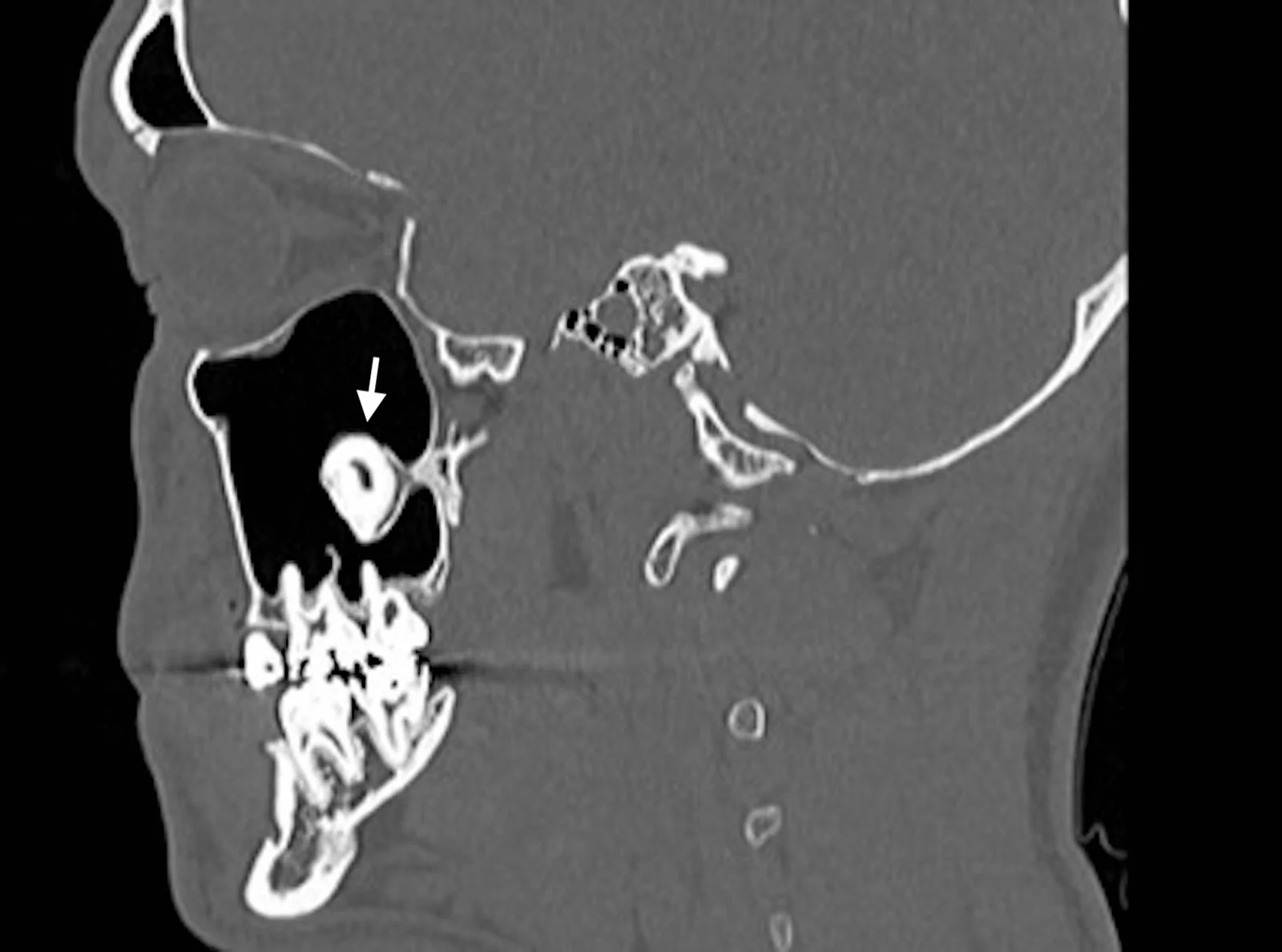
Preoperative sagittal section of a maxillofacial computed tomograph. An area of hyperdensity is noted in the antrum with an attachment to the posterior wall (arrow).

On the day of the operation, the patient underwent an uneventful nasotracheal intubation and was prepped and draped in the usual manner for the procedure. After appropriate dispersal of local anesthetic, teeth #16, 17, and 32 were removed without incident. Attention was turned to the right maxilla where a vestibular incision was made, and a full-thickness mucoperiosteal flap reflected to expose the anterior antral wall. The access window was drawn out with a marker, and a 0.8 mm KLS fixation plate was adapted over the planned window and medial and lateral portions of the antral wall. Fixation holes were pre-drilled in anticipation of replacing the bony window. The window was cut with a 701 bur and carefully removed and stored in normal saline. The FBITA was visualized with the use of a fiberoptic intra-antral endoscopic camera (Figure 3). The FBITA was removed entirely using a hemostat without incident. After debridement of the posterior sinus wall where the FBITA was anchored, the antrum was irrigated with copious normal saline. The bony window was affixed to the plate, which was subsequently fixated both laterally and medially, restoring continuity of the anterior antral wall (Figure 4). Bilayer collagen membranes were placed over the osseous surgical site, and the flap was closed primarily. The final histopathological analysis was that of an ectopic tooth. Postoperatively, the patient healed well without incident. 

**Figure 3:**
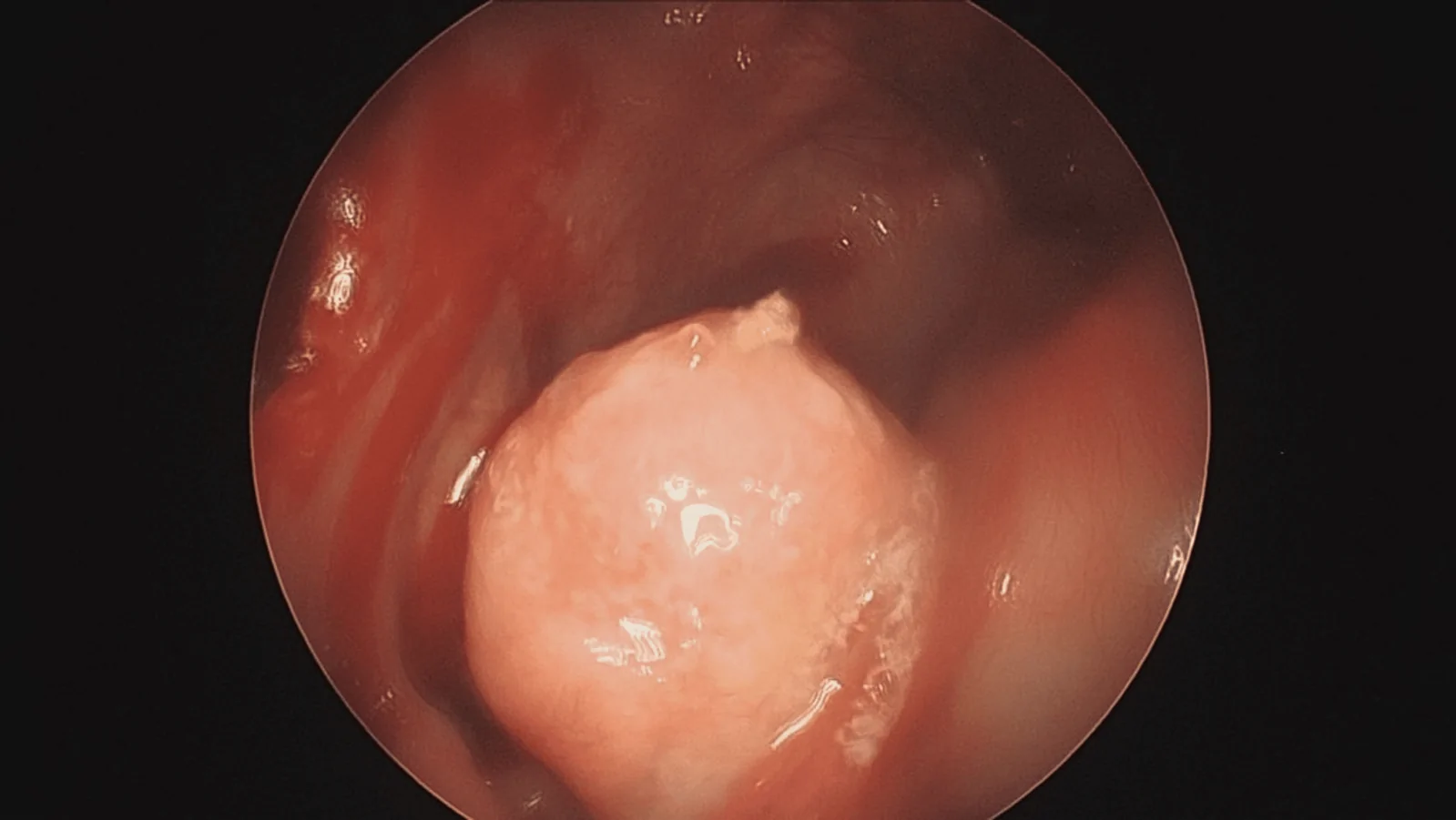
Image captured of the foreign body in the antrum (FBITA) in situ using the fiberoptic intra-antral endoscope.

**Figure 4:**
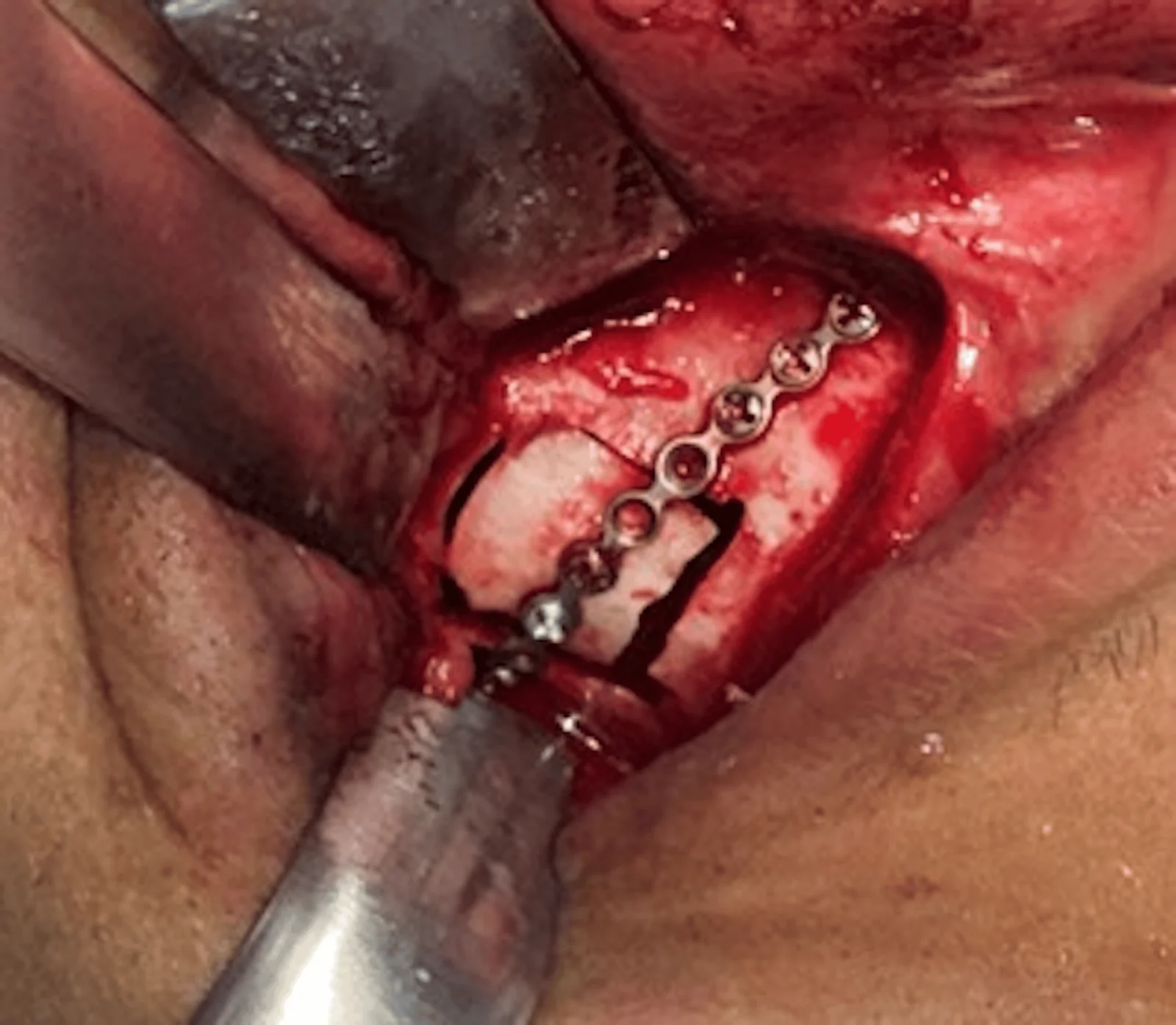
Image of the bony window re-plated, ensuring bony contact at two points.

Histopathologic findings

Histological examination demonstrated odontogenic structures composed of mature dentin, enamel, pulpal tissue, and associated dental follicular epithelium arranged in a relatively organized architectural pattern (Figure 5). The mineralized dental tissues maintained recognizable compartmentalization, with enamel and dentin distributed in distinct anatomic regions rather than haphazardly intermixed. Centralized pulp tissue was identified with a discernible pulp chamber-like space, and portions of the specimen exhibited evidence of organized root formation. Although the specimen lacked the conventional gross silhouette of a normally erupted tooth (Figure 6), the histologic findings supported a structured pattern of odontogenic development rather than a disorganized odontogenic proliferation.

**Figure 5:**
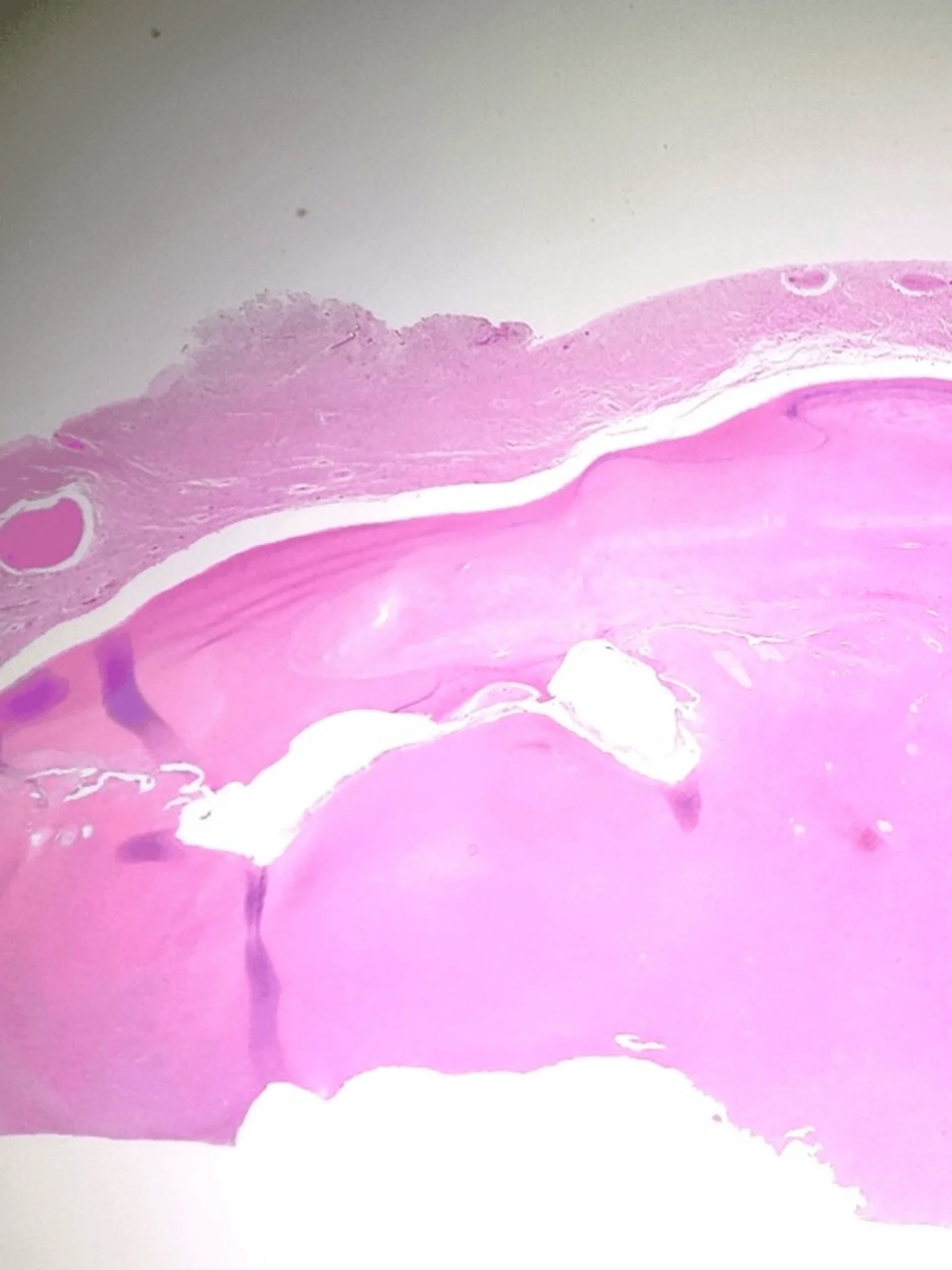
Low-power photomicrograph demonstrating relatively organized odontogenic architecture with mature dentin surrounding centrally located pulpal tissue and relatively well-defined root morphology.

**Figure 6:**
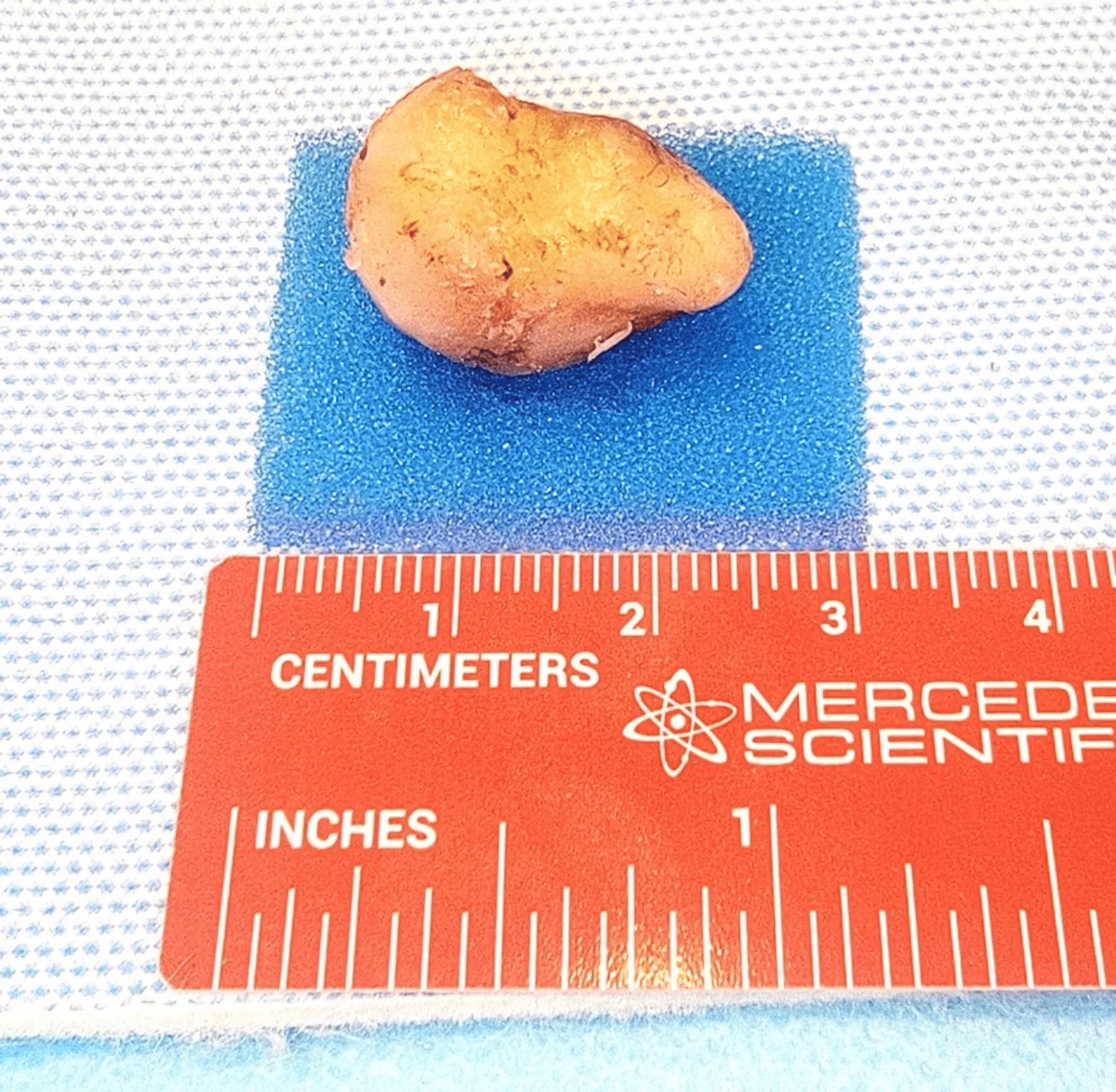
Gross photograph of the excised malformed ectopic tooth demonstrating an irregular external contour with atypical crown-root configuration.

Correlation with radiographic, intraoperative, and histopathologic findings provided valuable clinicopathologic context for interpretation of this case. Although the clinical differential diagnosis of odontoma was considered, the overall degree of tooth-like structural organization identified in the radiographic and histologic findings favored a malformed ectopic maxillary third molar over an odontoma.

## Discussion

Ectopic teeth in the maxillary sinus, particularly those affixed to the posterior wall, require careful preoperative imaging and consideration of surgical approach to ensure complete removal while minimizing complications [1,6]. This case demonstrates a unique variation on retrieval assisted with fiberoptic trans-antral endoscopy and preservation of the anterior antral wall in the oral and maxillofacial literature. 

A team in China devised a similar approach to treating their FBITA (an odontogenic keratocyst), although they elected to secure the bony window with resorbable suture [7]. At follow-up imaging, they noted that three of the four walls demonstrated bony healing [7]. In the absence of a midface plating system, or in the interest of saving cost to the patient, resorbable suture material may be a consideration for re-suspending the anterior antral window. An otolaryngology team from Iowa City employed both resorbable suture and semi-rigid fixation in the so-called bony lid approach to the antrum of Highmore to deal with various FBITAs [10]. They reported one patient who desired removal of the hardware two years after the initial surgery [10]. These two approaches, using both resorbable sutures and more robust plating, are other options the elegant surgeon can keep in the quiver of FBITA access and treatment.

## Conclusions

This case adds to the limited literature on ectopic teeth within the maxillary sinus. It illustrates a combined approach involving fiberoptic transantral endoscopy-assisted removal and restoration of anterior antral wall continuity. This approach highlights the potential value of endoscopic visualization alongside reconstruction of the surgical access site. The case may serve as an additional reference for oral and maxillofacial surgeons and otolaryngologists considering treatment options for FBITAs of varying etiologies.
